# Multilevel genome typing: genomics-guided scalable resolution typing of microbial pathogens

**DOI:** 10.2807/1560-7917.ES.2020.25.20.1900519

**Published:** 2020-05-21

**Authors:** Michael Payne, Sandeep Kaur, Qinning Wang, Daneeta Hennessy, Lijuan Luo, Sophie Octavia, Mark M. Tanaka, Vitali Sintchenko, Ruiting Lan

**Affiliations:** 1 School of Biotechnology and Biomolecular Sciences, University of New South Wales, Sydney, Australia; 2 Centre for Infectious Diseases and Microbiology–Public Health, Institute of Clinical Pathology and Medical Research – NSW Health Pathology, Westmead Hospital, Westmead, Australia; 3 Marie Bashir Institute for Infectious Diseases and Biosecurity, Sydney Medical School, University of Sydney, Westmead, Australia

**Keywords:** genomics, epidemiology, microbiology, molecular subtyping, outbreak detection, salmonella, nomenclature

## Abstract

**Background:**

Both long- and short-term epidemiology are fundamental to disease control and require accurate bacterial typing. Genomic data resulting from implementation of whole genome sequencing in many public health laboratories can potentially provide highly sensitive and accurate descriptions of strain relatedness. Previous typing efforts using these data have mainly focussed on outbreak detection.

**Aim:**

We aimed to develop multilevel genome typing (MGT), using consecutive multilocus sequence typing (MLST) schemes of increasing sizes, stepping up from seven-gene MLST to core genome MLST, to allow examination of genetic relatedness at multiple resolution levels.

**Methods:**

The system was applied to *Salmonella* *enterica* serovar Typhimurium. The MLST scheme used at each step (MGT level), defined a given MGT-level specific sequence type (ST). The list of STs generated from all of these increasing MGT levels, was named a genome type (GT). Using MGT, we typed 9,096 previously characterised isolates with publicly available data.

**Results:**

Our approach could identify previously described *S.* Typhimurium populations, such as the DT104 multidrug resistance lineage (GT 19-2-11) and two invasive lineages of African isolates (GT 313-2-3 and 313-2-752). Further, we showed that MGT-derived clusters can accurately distinguish five outbreaks from each other and five background isolates.

**Conclusion:**

MGT provides a universal and stable nomenclature at multiple resolutions for *S*. Typhimurium strains and could be implemented as an internationally standardised strain identification system. While established so far only for *S.* Typhimurium, the results here suggest that MGT could form the basis for typing systems in other similar microorganisms.

## Introduction

Accurate and reliable characterisation of bacterial pathogens is crucial for classifying related strains into clusters for both short- and long-term epidemiology. In short-term epidemiology, very similar strains are identified to facilitate the detection and tracking of specific outbreaks and disease transmission pathways to inform timely, effective interventions by public health authorities [1,2]. In long-term epidemiology, the tracking of bacterial clones, which may share characteristics such as antimicrobial resistance or increased pathogenicity, has been vital for understanding the population dynamics of pathogens [3,4]. Both are important to public health but have been performed using a plethora of separate, albeit related, tools with differing resolutions [5,6].

Multilocus sequence typing (MLST) has been used extensively to characterise bacterial populations at larger geographic and temporal scales [7]. This technique involves the examination of seven housekeeping gene fragments for variation. For a given strain, each of the seven fragments is assigned an allele number based on its unique nucleotide sequence, which may or may not be shared by other strains. The unique combination of all the fragments’ allele numbers, the allelic profile, is used to define a sequence type (ST). An ST is a stable and standardised identifier of a group of related strains, and numerous studies have used STs to track clones within a species that share important characteristics [8-10].

The deployment of whole genome sequencing (WGS) has the potential to establish a unified approach for both long- and short-term epidemiology. It has radically improved the ability to accurately and rapidly identify clusters of closely related isolates for detection of bacterial outbreaks. WGS can be used to delineate clusters with greater discriminatory power than traditional methods because the entire genetic complement of an organism is examined [11,12]. Many studies have used WGS, mostly through the comparison of single nucleotide polymorphisms (SNPs) across the genome, to cluster isolates and have had notable success in demonstrating their utility in outbreak detection [13,14]. Others have expanded the MLST concept from seven genes to include all loci in the core genome (core genome MLST (cgMLST)) or pan-genome (whole genome MLST (wgMLST)) of a species [15,16]. cgMLST and wgMLST have also proved useful for the identification of clusters and investigation of outbreaks [17-19].

MLST-based methods characterise the relatedness of strains based on the allelic profile differences between those strains [20,21]. The MLST approach has been referred to as gene-by-gene comparison in contrast to SNP-based comparison. For closely related strains, a gene difference may only contain one SNP and the two approaches achieve similar resolution [22]. However, this resolution can be too high for monitoring clones over longer time frames. There is therefore a need for systems that can classify isolates at different resolutions to cover the spectrum between MLST and cgMLST. One approach to solve this problem is to use hierarchical clustering using either SNPs or allelic differences to generate classification systems at multiple resolutions such as SNP address and HierCC [23,24]. These systems provide accurate clustering but suffer from issues such as a lack of stability and founder effects when additional data are added.

Here, we present a novel system of MLST schemes with a gradient of resolutions that facilitate the stable comparison of strains within a species at multiple levels of relatedness. We refer to the method as multilevel genome typing (MGT). We demonstrate the application of MGT using *Salmonella enterica* serovar Typhimurium (STM), which is the single largest cause of salmonellosis in Australia and one of the most prevalent serovars in many other areas of the world including Europe [25].

## Methods

### The multilevel genome typing concept

#### Multilocus sequence typing schemes and long- vs short-term epidemiology

**Figure 1:**
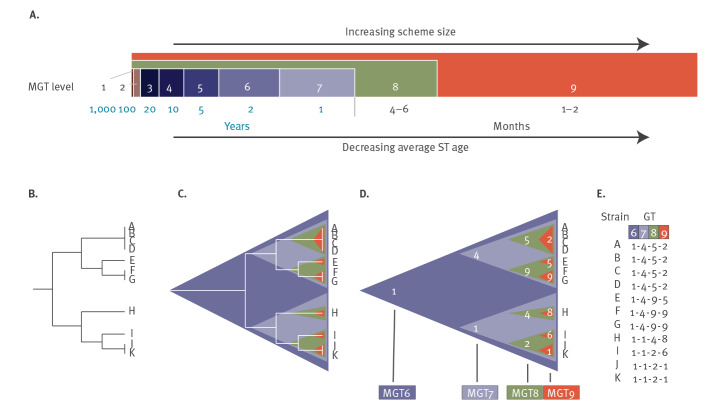
Structure and concept of the multilevel genome typing (MGT) system

The classic seven-gene MLST scheme defines STs that represent long-lived and historical clones while cgMLST schemes can define STs that capture clones that have arisen recently. The former scheme has very low resolution while the latter scheme has very high resolution. We propose a set of methodologically connected MLST schemes, individually called levels, that offer a gradient of resolutions for the comparison of isolates (Figure 1A). The assignment to each of these levels allows the study of long-term, and short-term (outbreak) epidemiology while providing each isolate with an identity defined by a series of STs (each defined by separate allelic profiles) from each of the levels (smallest to largest). This string of STs is named a genome type (GT), the method is called MGT and the overall system for a given organism is called an MGT scheme.

cgMLST: core genome multilocus sequence typing; GT: genome type; MGT: multilevel genome typing; MLST: multilocus sequence typing; ST: sequence type.

A. Schematic representation of the nine MLST schemes that make up the MGT levels. The increasing number of loci in each level is represented by increasing length of the coloured horizontal bars. MGT1 is the classic seven-gene MLST scheme. MGT levels 1 to 7 are mutually exclusive and are made up of a subset of MGT8 (cgMLST). MGT9 is made up of all loci including intergenic regions and encompasses all other levels (cgMLST plus clade-specific cgMLST). MGT levels are ordered by size, and loci are selected, so that each level matches the desired size, which confers the desired average mutation rate.

B–E. The relationship between phylogenetic relatedness and the MGT.

B. An example phylogeny depicting the relationship between 11 strains.

C. MGT6–9 STs overlaid onto the phylogeny. Each ST for each MGT level is represented by a triangle with the colour corresponding to the MGT level. As levels increase in resolution (increase in number of loci) smaller subclades are defined by each ST.

D. Example STs for MGT 6–9 are overlaid onto each clade.

E. The relationship between isolates expressed only as MTG 6–9 STs. The relatedness of a group of isolates is represented by the largest level that they share an ST (e.g. isolate A and B share an MGT9 ST and so are more closely related than A and E which only share an MGT7 ST).

#### Multilevel genome typing levels and their size

The number of MGT levels and size (number of nucleotides) of those levels for a given MGT scheme depend on the resolution required and the genetic diversity of the population of the individual species or clones. In order for each level to have adequate resolution for epidemiological tracing of clones of various longevity, evolutionary rate was used to define level sizes (Supplementary Methods). Each level was generated so that an allelic difference in one of the loci in the level will, on average, occur once in a defined time period. To achieve this, we used the mutation rate of a given species to model the time required for a mutation to arise in a given length of sequence. We then selected loci so that the combined sequence length for each level resulted in the expected rate of new alleles. For STM, we define levels that can describe molecular epidemiology over a range of evolutionary time spans. Average rates of one allele change per 100, 20, 10, 5, 2 and 1 year were defined for MGT2, 3, 4, 5, 6 and 7 levels and resulted in sizes indicated in the Table. It should be noted that the average rate of a given level does not necessarily reflect the age of a given ST as the range of ages is likely to be large. The two largest levels, MGT8 and MGT9 represent the species and serovar cgMLST, respectively. These two levels offer the highest resolution and can be used for outbreak detection and identification. The classical seven-gene MLST scheme may be included as the first level (MGT1) for backward compatibility. Therefore, for STM, we define a nine level MGT scheme.

**Table:** Multilevel genome typing summary statistics with application to 9,096 *Salmonella* Typhimurium genomes

MGT LEVEL	LOCI	TOTAL LENGTH (KB)	PROPORTION OF LT2 GENOME SIZE (%)	AVERAGE NUMBER OF ISOLATES PER ST	NUMBER OF STS FROM 9,096 GENOMES
**MGT1^a^**	7	3.3	0.07	115	74
**MGT2**	18	10.8	0.22	37.43	256
**MGT3**	77	53.2	1.10	8.25	1,162
**MGT4**	156	105.6	2.17	4.59	2,110
**MGT5**	241	210.4	4.33	2.94	3,296
**MGT6**	682	525.8	10.82	1.87	5,188
**MGT7**	1,044	1,051.6	21.67	1.48	6,626
**MGT8**	2,956^b^	2,788.1	57.40	1.24	7,926
**MGT9**	5,293^c^	4,013.3	82.62	1.17	8,425

LT2: reference LT2 genome (GenBank number: NC_003197.2); MGT: multilevel genome typing; MLST: multilocus sequence typing; ST: sequence type.

^a^ Classical seven-gene MLST scheme.

^b^ *Salmonella* core genes.

^c^ *S.* Typhimurium core genes and core intergenic regions.

For complete lists of loci see Supplementary Methods Table 4.

#### Nomenclature of genome types in multilevel genome typing

Flexible notations of the naming system further enhance the utility of MGT. For example, a full GT consisting of nine ST numbers separated by hyphens gives the precise definition of a strain (GT 19-2-11-27-115-274-365-435-501). A partial GT can be used to describe a broader set of isolates (GT 19-2-11-27-115-274-X-X-X) with X representing an undefined ST at a given MGT level. This may be shortened to GT 19-2-11-27-115-274. An ST at any MGT level may also be used to define a clone (MGT4 ST27), similar to traditional MLST. Finally, in situations where multiple related GTs need to be communicated, a degenerate GT can be used (GT 19-2-11-(27/32)-115-274-365-435-501).

#### Genome types and phylogenetic relationships

The STs defined by the gradient of MGT levels also define relationships between GTs that approximate phylogenetic relationships. The lower resolution levels produce STs that are mostly subdivided by the next level with higher resolution. This process continues with division of isolates into progressively smaller clades. In this way, the relationships of GTs to others are visible at multiple scales simultaneously and approximate the branching of the phylogenetic tree (Figure 1B-E) albeit with some important, but resolvable, exceptions. Importantly, the loci used in each level are mutually exclusive with the exception of the two largest levels (MGT8 and MGT9). These mutually exclusive levels offer the opportunity to infer evolutionary relationships independently. The main caveat is that a mutation in a lower resolution level (e.g. MGT3) could result in two separate STs while a higher resolution level (e.g. MGT4) has one ST. This situation is defined as a hierarchical inconsistency and is the result of random mutation locations and the non-overlapping nature of the levels. However, this hierarchical inconsistency can be resolved by interrogating levels above and below the inconsistent one, allowing the true relationship between isolates to be described.

We further implemented clonal complexes (CC) for MGT STs at each level, which are defined by a group of STs that differ by one locus from any other ST in the complex [26]. These CCs can reveal additional groups of related isolates and can allow the identification and resolution of hierarchical inconsistencies. However, CCs can merge when an isolate is equally close to two previously separate clonal complexes. This means that CC assignments can change with the addition of isolates and as such they cannot be used as a stable isolate nomenclature and should only be used to aid interpretation of GTs and resolve hierarchical inconsistencies.

#### Outbreak detection clusters for flexible and high-resolution investigations

To facilitate outbreak detection, we use MGT9 to identify potential outbreak clusters as MGT9 has the highest resolution among all the levels. No uniformly applicable cluster cutoffs have been established for outbreak detection. We therefore define potential outbreak detection clusters (ODC) using multiple cutoffs for the maximum number of allele differences. Potential outbreaks can then be identified using additional parameters such as spatial and temporal information. An ODC is defined by a given number of locus differences. For example, all isolates within one ODC5 cluster are less than or equal to five allelic difference from at least one other isolate in the cluster. Four levels of ODCs were implemented with clustering cutoffs at 1, 2, 5 and 10 loci differences, named ODC1, ODC2, ODC5 and ODC10, respectively. These ODCs were used to explore the impact of resolution on the identification of clusters. However, GTs not ODCs should be used for naming outbreak causative strains due to the instability of ODCs when new isolates are added. ODCs are provided for detecting potential outbreak clusters.

### Defining multilevel genome typing levels for *Salmonella* Typhimurium

Applying MGT to STM, the target size of each MGT level was determined using the average mutation rate of STM and the desired new allele generation rate for each level (e.g. every 2 years) (Supplementary Methods). Loci in the Enterobase cgMLST scheme (3,002 loci) were compared with the 5,478 core genes and intergenic regions found in the STM core [12,27]. Loci that were reliably callable in less than 96% of 9,096 genomes tested were removed from the MGT entirely and these loci were considered untypeable. This threshold is slightly more stringent than the Enterobase cgMLST scheme, which used a 94% cutoff [27]. After removing the untypeable loci, 2,956 loci were retained to generate the MGT8 level while 5,293 loci were used in the MGT9 level.

Filters applied to select most favourable loci for the smallest levels were as follows. A locus could be included in MGT2, 3 and 4 if it was never called as missing or partially missing in a dataset of 9,096 genomes investigated in this study, for MGT5 and 6 the inclusion criterion for a locus was relaxed to allow a maximum of five genomes missing or partially missing it and subsequently, for MGT7 this was further relaxed to 25 genomes. It should be noted that even at the maximum cutoff of 25 (of 9,096 genomes) only 0.27% of genomes would contain missing data for a given locus. The average number of partially missing (< 20% absent) and missing (> 20% absent) loci per isolate per level was measured to ensure only high quality loci were used (Supplementary Figure 3). In order to ensure that strong positive or negative selection was not occurring in loci in the smaller levels, the ratio of non-synonymous and synonymous substitutions (dN/dS) for each locus was obtained from a previous study [28] and their distribution was taken into account. Loci with dN/dS between the 25^th^ and 75^th^ percentiles were initially used in MGT2 followed by loci from the 5^th^ to 95^th^ percentiles for MGT 3–7. An Enterobacteriaceae core was defined using 20 species (Supplementary Table 6) using Roary ([29], v3.12) with sequence identity of 70% and presence proportion of 100% and included 1,540 loci. Only loci from this core were included in MGT2 and MGT3. We also selected loci for MGT2, MGT3 and MGT4 with cytoplasmic subcellular localisation as defined by a combination of Biocyc and PSORT classifications as well as predictions from SignalP (version 4.1) and TMHMM (version 2.0c) [30-33], in order to select genes that were not likely to be interacting with the host and therefore not likely to be under positive selection. Genes matching the following criteria were also excluded from the levels indicated: genes in functional categories of ‘virulence’, ‘virulence, disease and defence’, ‘cell wall and capsule’, ‘phages, prophages, transposable elements’ and ‘motility and chemotaxis’ [34] (MGT2–4); known or predicted effector proteins [28] (MGT2–6); genes within prophages, detected using PHAge Search Tool (PHAST) and PHAge Search Tool Enhanced Release (PHASTER) [35,36] (MGT2–6); genes containing tandem repeats, identified with tandem repeat finder (TRF, version 4.09) [37] (MGT2–6); genes containing homopolymers, identified with an in house Python script (MGT2–6). Combinations of filters were applied with decreasing stringency from MGT2 to 7 as listed in Supplementary Table 7. These filter combinations were named preferences. The smallest preference number denoted the combination of filters that is most stringent. The combinations of largest preference number allowed and minimum distance between loci for each MGT level are listed in Supplementary Table 8 as well as the numbers of loci with each preference in each level. Loci assignments to each level are listed in Supplementary Table 9. Genome location of loci in MGT levels was displayed using Basic local alignment search tool Ring Image Generator (BRIG) [38].

### Genome data

Raw read data were downloaded from the European Nucleotide Archive and their species confirmed using Kraken version 1.1.1 [39]. The shovill pipeline (http://github.com/tseemann/shovill) was then used to process (Trimmomatic, Lighter, Fast Length Adjustment of SHort reads (FLASH)) and assemble (strategic k-mer extension for scrupulous assemblies (SKESA)) reads, as well as remap reads for error correction (Burrows–Wheeler Aligner (BWA), Pilon) [40-45]. Genome quality was then assessed using Quality Assessment Tool for Genome Assemblies (QUAST; version 5.0) and serotype was confirmed using *Salmonella* In Silico Typing Resource SISTR (version 1.0.2) [46,47]. Genomes passing previously defined filters were used for further analysis: < 700 contigs, largest contig > 60 Kb, genome length between 4.5 and 6 Mb, GC percentage between 50 and 54, N50 > 50 kb, gene number > 3,000 [48].

### Genome type, clonal complex and outbreak detection cluster calling

Allele calling was performed using nucleotide-nucleotide basic local alignment search tool (blastn; v2.6.0) and Python scripts, which make up part of the MGT analysis pipeline (Supplementary Methods). ST and GT calling as well as CC and ODC clustering were performed with further MGT analysis pipeline scripts (Supplementary Methods). MGT calls, CCs and ODCs for DT170, DT160 and DT104 can be found in Supplementary Tables 1–5. An MGT database for STM is available to facilitate visualisation and exploration of the over 9,000 isolates examined here (http://mgtdb.unsw.edu.au) and can be used for further data submission.

### Phylogenetic tree construction and visualisation

The phylogeny for 9,096 STM genomes was generated from MGT allele profiles using Grapetree [49] with the ‘-m RapidNJ’ parameter. Visualisation was performed using Grapetree’s interactive mode. For DT170 isolates, a SNP alignment was generated from MGT9 alleles relative to alleles derived from the LT2 reference genome. This alignment was used to generate a phylogeny using minimum evolution as implemented in Molecular Evolutionary Genetics Analysis (MEGA) version 7 [50].

## Ethical Statement

Ethical approval was not required for this study.

## Results

### Implementation of multilevel genome typing for *Salmonella* Typhimurium

**Figure 2:**
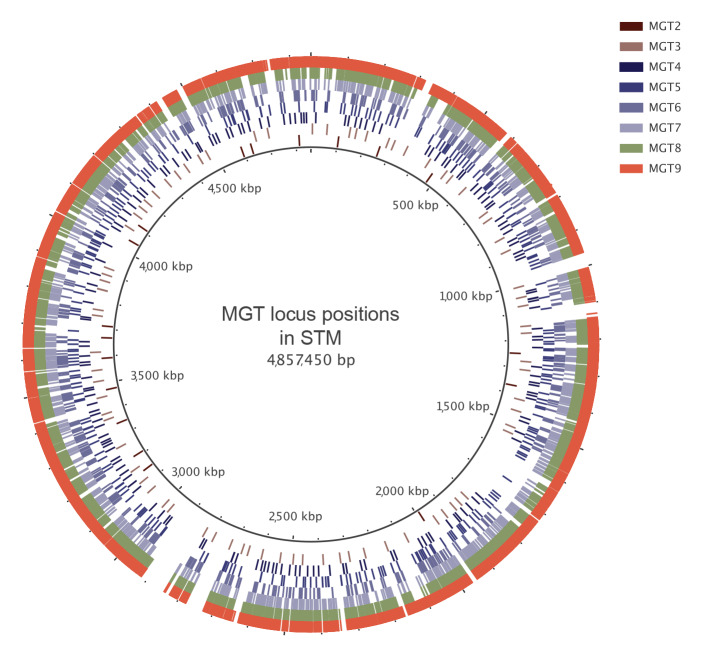
The positions of loci assigned to each of the eight generated multilevel genome typing levels for *Salmonella* Typhimurium

MGT: Multilevel genome typing; STM: *Salmonella* Typhimurium.

As introduced above, STM MGT scheme consists of nine levels, the highest resolution level, referred to as MGT9, uses all STM core genes and intergenic regions [12]. The STM core covers 83% of the reference LT2 genome (GenBank number: NC_003197.2). MGT8 consists of 2,956 *Salmonella* core genes. This level overlaps with the *Salmonella* cgMLST scheme implemented in Enterobase [27], with the omission of 53 loci due to their small size or unreliability in assembly. MGT1 is the classical seven-gene *Salmonella* MLST scheme [8]. The MGT 2–7 levels consist of between 18 and 1,044 loci from the *Salmonella* core gene set (Table) and are fully described in Supplementary Tables 2–4. The selection of loci is described in Supplementary Methods. The genomic positions of loci in each level are separated by a minimum distance to reduce the impact of recombination (Figure 2).

Positions are for the STM reference genome LT2 (GenBank number: NC_003197.2). The minimum spacing of two loci in a given level was 20 kb for MGT2 and MGT3, 8 Kb for MGT4, 4 kb for MGT5 and 0 Kb for MGT6 and 7. These distances also include loci from the previous levels. For example, all loci in MGT4 are greater than 8 Kb away from loci in MGT2, 3 and 4. The number of loci included in MGT2 to MGT9 are as follows: MGT2 18; MGT3 77; MGT4 156; MGT5 241; MGT6 682; MGT7 1,044; MGT8 2,956 (*Salmonella* core), MGT9 5,293 (STM core).

**Figure 3:**
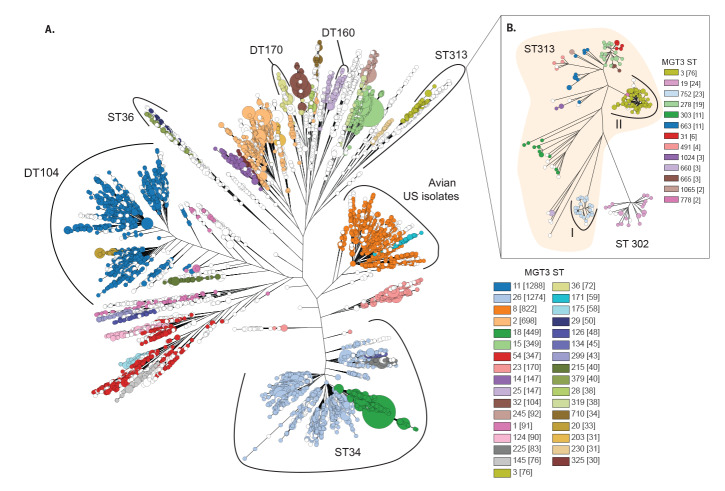
*Salmonella* Typhimurium population structure obtained with multilevel genome typing (MGT) level 3 sequence types (n = 9,096 genomes)

MLST: multilocus sequence typing; ST: sequence type; US: United States.

The nine MGT levels were used to assign GTs to 9,096 STM genomes (Table). MGT9 allele profiles were also used to calculate a phylogenetic tree, which was labelled with MGT3 STs (Figure 3A). At MGT3 level 77% (7,017/9,096) of the isolates were represented by STs that contained more than 30 isolates. These included DT104, DT160, an Australian DT170 lineage and a cluster of United States (US) avian isolates, which can be identified by their unique MGT3 STs. For the seven-gene MLST (MGT1) ST313 that causes invasive infections in Africa, the two previously identified invasive lineages can be almost entirely identified by single MGT3 STs (ST752 for lineage I and ST3 for lineage II) [51] (Figure 3B). These two invasive lineages can also be distinguished from other ST313 isolates, identified in the United Kingdom (UK), which mostly cause gastrointestinal disease and are classified into at least nine other MGT3 STs [52]. This demonstrates the utility of the MGT to describe isolates at more than one resolution. MGT3 as an example, and the MGT as a whole, is capable of providing stable identifiers for clinically relevant groups of isolates, which were previously only discernible through construction of phylogenetic trees.

A. A dendrogram of 9,096 isolates derived from the allelic profiles of the largest MGT9 MLST scheme. STs with more than 30 isolates were marked. Branch lengths are log scale for clarity. Phage types and seven-gene MLST STs of interest are marked. One clade of isolates is almost entirely composed of isolates derived from birds in the United States and is identified by MGT3 ST8 and ST171. DT104 is identified by MGT3 ST11, seven-gene MLST ST34 (monophasic Typhimurium) is identified by MGT3 ST26, ST18 and ST225.

B. A subtree derived from section A showing seven-gene MLST ST313 as well as its closest outgroup ST302. Previous studies have identified two lineages of ST313 that cause invasive salmonellosis. These lineages, I and II, are shown and can be defined by MGT3 ST752 and MGT3 ST3 respectively. Importantly MGT3 can distinguish both invasive lineages from other ST313 isolates that do not cause invasive disease.

Numbers in square brackets denote the number of isolates assigned each ST.

The degree of hierarchical inconsistency was examined in all STM genomes with GTs. The highest percentage was 17.5% in both MGT3 and MGT4 with all other levels below 10% (Supplementary Figure 2). The number of levels with hierarchical inconsistency in each isolate was also examined: 51.2% of isolates had no inconsistent levels, 40.5% had one, 7.8% had two and 0.5% had three. Therefore, in all isolates the majority of MGT levels are consistent and should provide meaningful evolutionary relationships.

### Application of multilevel genome typing to long-term epidemiology: *Salmonella* Typhimurium DT104 as an exemplar

**Figure 4:**
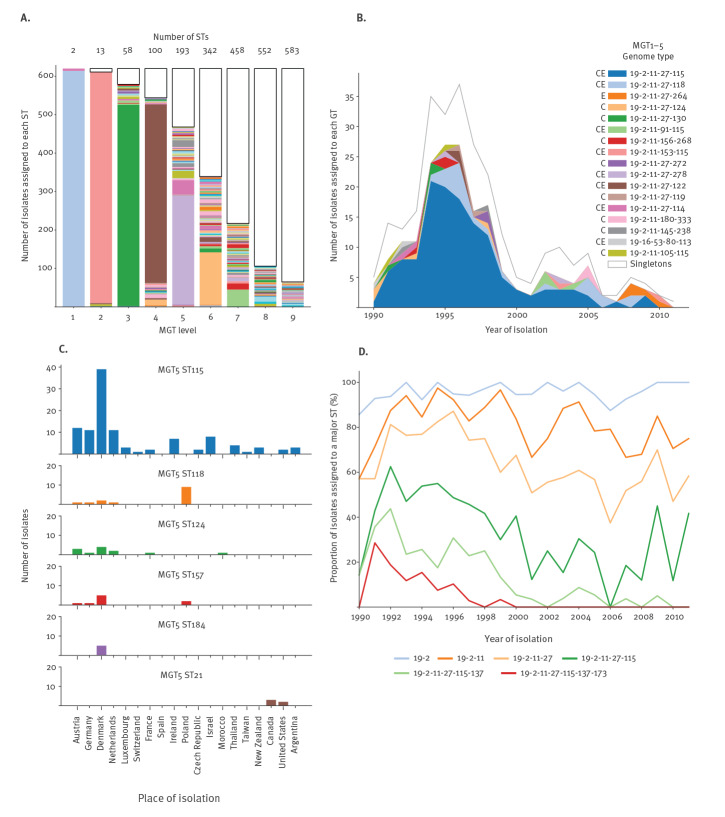
The multilevel genome typing describes temporal and spatial trends and clusters in *Salmonella* Typhimurium phage type DT104 (n = 619)

The intermediate levels (MGT2–7) allow the examination of larger spatial and temporal population trends. These levels were used to examine the population dynamics of DT104 over more than 20 years from more than 20 different countries using 619 DT104 isolates from two previous studies [13,53]. The number and proportion of STs called at each of the nine levels are shown in Figure 4A. In low resolution levels (MGT1 and MGT2), the majority of isolates are assigned one ST while in the higher resolution levels (MGT7, 8 and 9) the majority of isolates are assigned to STs with only one or two other isolates. This demonstrates the diversity present within DT104 as well as its single origin.

C: clinical source (panel B); E: environmental source (panel B); GT: genome type; MGT: multilevel genome typing; ST: sequence type; UK: United Kingdom.

A. 619 DT104 isolates assigned to STs at each MGT level. STs with two or more isolates are grouped into coloured blocks while STs assigned to only one isolate are grouped into one category shown as the white box with black outline. The number of STs identified for each scheme is shown above each column.

B. Temporal distribution and source type of MGT1–MGT5 GTs in the UK from 1990 to 2011. 273 isolates used in Mather et al. (2013) [13] that were isolated in the UK were classified using GTs. The number of isolates were distributed according to their year of isolation and stratified by partial GTs including MGT levels 1 to 5. C denotes clinical and E denotes environmental sources. If both are present, strains annotated with the GT come from both sources. If only one is shown all strains annotated with the GT come from that source type. GTs occurring in one isolate were grouped together as ‘singletons’ for clarity.

C. Geographical distribution of MGT5 STs. 289 strains used in Leekitcharoenphon et al. (2016) [53] were classified using MGT5. MGT5 STs occurring in more than three isolates are shown. MGT5 ST115 is by far the largest group and is the most common ST in most countries. MGT5 ST118, ST124 and ST157 are generally restricted to north-western Europe and Poland. MGT5 ST184 is restricted to Denmark while MGT5 ST21 is restricted to North America.

D. The proportion of 619 isolates sequenced in Leekitcharoenphon et al. (2016) and Mather et al. (2013) [13,53] assigned to the most common MGT2–7 GTs over time. The major GT for each level was determined and the percentage of isolates in a given year classified into that GT is shown. With the exception of MGT2 and MGT3, all other MGT levels show a reduction in the major type over time.

The 273 DT104 isolates from Mather et al. [13] that were isolated in the UK over a period of 21 years were used to examine the usefulness of the MGT in examining temporal population changes (Figure 4B, Supplementary Table 1). Most isolates were assigned to GT 19-2-11-27 (235/273). At one level higher (MGT1–5), GT 19-2-11-27-115 was the most prevalent, containing 49% (135/273) of the isolates, and was found across the entire timespan of the data represented and in both environmental and clinical strains. Several patterns were observed among the remaining minor GTs. GT 19-2-11-27-118 was first observed in 1994 and was still observed 14 years later suggesting that it existed as a minor subpopulation alongside the major GT 19-2-11-27-115. GT 19-2-11-27-124 was similar however it was only detected for 8 years. The majority of the remaining GTs were short lived and found only in clinically derived strains suggesting that they may represent single outbreaks (including GT 19-2-11-27-130, 19-2-11-27-272, 19-2-11-27-119, 19-2-11-180-333, 19-2-11-105-115). GT 19-2-11-27-264 is restricted to environmental samples and only emerged in 2008. These results show that MGT can track the frequency of GTs over time allowing emerging or clinically important strains to be monitored both locally and globally.

The utility of the MGT to examine the spatial distribution of DT104 GTs was examined using 289 isolates from Leekitcharoenphon et al. [53] (Figure 4C, Supplementary Table 2; note that here the simple MGT level notation was used). MGT5 ST115 was the most common MGT5 type and made up the majority of DT104 isolates in most countries. MGT5 ST118 was predominantly found in Poland from 2000 to 2011 suggesting that this type may have a local reservoir in that country. Similarly, MGT5 ST157 was identified in Denmark over a 10-year period. There is also evidence of a North-America-restricted type with MGT5 ST21 only isolated from Canada and the US over a 12-year period. This agrees with Leekitcharoenphon et al. who identified the same North American clade using phylogenetic analysis.

As illustrated in Figure 4D with 619 isolates [13,53], the temporal population structure of DT104 is also captured by the MGT as strains diverge from each other. This diversification leads to increasingly dissimilar isolates over time (Figure 4D, Supplementary Table 3). MGT is able to capture this temporal variation because the larger the MGT level the less time an ST derived from it is expected to have existed. This can be seen in MGT levels 5, 6 and 7. At MGT5, GT 19-2-11-27-115 reduced from 46.5% (127/273) of the isolates throughout the 1990s to 21.8% (75/344) in the 2000s. At MGT6, GT 19-2-11-27-115-137 made up 24.5% (67/273) of the isolates from the 1990s had almost disappeared by 2011. Finally, at MGT7, GT 19-2-11-27-115-137-173 which was the most common in 1991 was not sampled after 2000. When MGT was applied, a similar pattern of diversification was also observed in a point source epidemic of phage type DT160 in New Zealand [54]. Over the 14 years of the study the major GT 19-5-25-163-292 reduced from 100% of isolates in the first 2 years to a minority in the last 4 years (Supplementary Figure 1, Supplementary Table 4). The DT104 and DT160 datasets demonstrate the utility of MGT in describing long-term spatiotemporal trends of clinically important clades.

### Application of multilevel genome typing to outbreak detection and strain attribution

**Figure 5:**
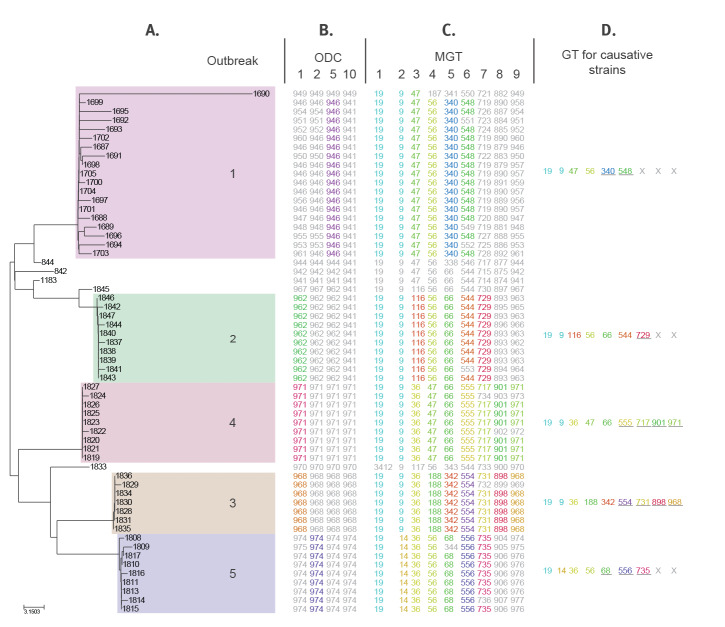
Multilevel genome typing applied to five outbreaks and background DT170 strains (n = 59)

GT: genome type; MGT: multilevel genome typing; ODC: outbreak detection cluster.

ODCs were used to re-examine five epidemiologically confirmed point source outbreaks reported previously [11] (Figure 5, Supplementary Table 5). Outbreaks 2, 3 and 4 were detected at ODC1 level with causative isolates grouped into ODC1 clusters 893, 898 and 901 respectively. Outbreak 5 isolates formed cluster 974 at ODC2. Outbreak 1 was more diverse and as such its isolates formed cluster 946 at ODC5. One isolate from outbreak 1 was distant from the main outbreak ODC, which reflects the multi-strain nature of this outbreak as previously reported [11].

A. Minimum evolution phylogenetic tree depicting the relationships between the five outbreaks and background isolates from Octavia et al. (2015) [11].

B. ODC clusters with 1,2,5 and 10 allele difference cutoffs applied to the 59 isolates. Highlighted ODC levels and numbers indicate the smallest cutoff ODC level capable of describing all isolates in an outbreak. For outbreaks with closely related isolates (outbreaks 2,3 and 4) ODC1 was sufficient. However, for outbreaks with more divergent strains ODC2 (outbreak 5) or ODC5 (outbreak 1) was required to capture all outbreak isolates.

C. Full GT type for each isolate.

D. Potential outbreak strain naming. Once the outbreak was identified (and confirmed using epidemiological or other parameters) the GT of causative strains can be assigned. Examples of strain naming given for different outbreaks ranged from a full GT to partial GT notations. This naming facilitates public health communication as well as epidemiological surveillance. Underlined STs within each GT denote STs unique to that outbreak.

Once the outbreak is identified by ODCs, the implicated strains can be assigned a full or partial GT that names them for strain attribution and reporting. The flexibility of the MGT means that for different outbreaks, different levels can be used. For example, outbreak 3 isolates were assigned GT 19-9-36-188-342-554-731-898-968. For more diverse outbreaks, this identifier may not include all MGT levels. For example, outbreak 1 isolates can be described as GT 19-9-47-56-340-548. In cases where not all isolates shared identical GTs at all levels we assigned a GT to the causative strains where greater than 75% of the isolates shared the GT. A degenerate GT could also have been defined but was not included here for clarity. Therefore, ODCs can assist in the identification of an outbreak and GTs can give the causal strain a standardised name that can be used for epidemiological purposes and public health communication, with flexibility to accommodate minor variants.

## Discussion

The approach outlined and implemented in this study fills a critical gap in the implementation of genomic epidemiology of STM and could be applied to many bacterial pathogens. It also addresses the urgent need for standardisable strain nomenclatures [1,55]. MGT is composed of a series of STs derived from a series of MLST schemes or ‘levels’ that increase in the number of loci included from the classical seven-gene MLST scheme to the largest cgMLST scheme containing 5,293 loci (3,874 genes and 1,419 intergenic regions). MGT provides a means to identify clones at a resolution appropriate to the population being examined from short- to long-term epidemiology. In combination, the lower resolution levels allow for longer-term epidemiology over years or decades as well as global epidemiology, whereas the higher levels use cgMLST to provide a resolution that is capable of identifying very closely related isolates for outbreak detection. We have developed MGT for STM and have demonstrated its utility. STM is relatively low in diversity and can be used to best illustrate the usefulness of different levels of MGT for both short- and long-term epidemiology.

One of the advantages of MGT is that it provides clone and strain level nomenclature. MGT provides different levels of identity and thus allow identification of clones of different longevity from thousands of years in MGT1 to months in MGT9. Since the levels of the MGT are independent and increase in resolution, they provide flexibility to trace identities of clones or GTs over time. MGT is based on exact matching at each level and therefore avoids issues that single linkage clustering based methods (e.g. HierCC and SNP address [23,24]) can encounter, such as founder effects or cluster merging. These issues become more pronounced in the context of ever-expanding databases where the increase in isolates sampled can bridge the gap between previously separate clusters. STs and GTs assigned are expected to be stable despite the addition of large amounts of new isolates, which is likely to be very relevant as the world adopts genome sequencing for public health epidemiology. This likely increase in data also highlights the reason that specific mutations, selected from predefined phylogenetic trees or populations, were not used to select loci when constructing MGT levels in this case. The addition of potentially novel clades in the future means that schemes selected in such a way may not be universally useful. The MGT in its current form avoids this issue by ignoring the existing population structure when selecting loci. Therefore, MGT is envisaged as a standardised nomenclature that is stable and long lasting regardless of the size or structure of the population described. However, in cases where the population structure of the pathogen is very well defined and thoroughly investigated selecting loci that reflect the population structure may be more beneficial. Regardless, the multiple level MGT nomenclature offers flexibility of communication of strain identities and is useful for the communication and annotation of basic strain relationships.

The capabilities of MGT in tracking isolates for longer-term epidemiology were illustrated by DT104 and ST313. The former has been extensively studied previously with a relatively large set of genome data over different years and geographical regions [13,53]. DT104 emerged in the early 1990s and spread across the globe. We show that the DT104 isolates are easily identifiable using MGT. GT 19-2-11 defines DT104 and the higher resolution levels can further define the clinically restricted group GT 19-2-11-27-124 within DT104. At the same time, the highest levels identified closely related as well as temporally and spatially restricted groups of isolates, such as GT 19-2-11-27-115-274-365-435, which was identified only from pigs in Denmark in the year 2000. Similarly, two lineages (I and II) of ST313 causing invasive extra-intestinal infections in Africa that had only previously been described through phylogenetic analysis can now be broadly represented by MGT3 ST752 and MGT3 ST3, respectively [51,52]. These lineages arose independently from a larger ST313 background population, which is composed of mostly non-invasive isolates with different MGT3 STs. These results further demonstrate the usefulness of the multi-level nature of the MGT.

MGT also provides meaningful relationships between GTs as the scheme provides a gradient of resolution. Large STs defined by the lower resolution MGT levels (e.g. MGT3) can be divided into multiple smaller STs in the higher resolution MGT levels (e.g. MGT7). Figure 3 demonstrates this relationship between seven-gene/MGT1 STs and MGT3 STs, which exemplifies the sequential nature of the MGT as a whole. This relationship is very beneficial in recognising clones that are circulating locally or globally for epidemiological purposes. The MGT allows rapid communication of not only GT information but also the stable relationships of the isolates in a very simple format for public health investigations.

MGT is designed to be deployable for outbreak investigations and support outbreak case definitions. The focus of most studies into the utility of WGS has been in outbreak identification and investigation. The STM MGT scheme has the highest resolution in MGT9, which consists of an STM-specific cgMLST that includes STM specific core genes and core intergenic regions and is 25% larger than the species core genome. We have shown previously that the STM core genome offers higher resolution than the *Salmonella* core genome for STM outbreak investigation [12]. SNP/allele cutoffs for outbreaks vary as distance of background isolates to outbreak isolates depend on evolutionary time, mutation rate and local population diversity. We address this problem by providing four cutoffs (1, 2, 5 and 10) as ODCs based on MGT9 that allow the user to choose the appropriate SNP/allele difference cutoff for each outbreak scenario in the local context. These ODC were able to identify five STM outbreaks from Octavia et al. (2015) [11] that were distinct from each other and closely related background isolates.

ODCs are conceptually equivalent to HierCC [24] and are also similar to SNP addresses [56] except in cases where alleles differ by more than one SNP. Because ODCs suffer from stability issues common to all clustering methods they are envisaged to be used primarily for outbreak identification, not outbreak naming. This outbreak identification step is an area of active research and it should be noted that we do not demonstrate a method for selecting which ODC level is best. Rather we show that ODCs are capable of capturing the varying diversity of known outbreaks, which would be followed by naming of the causative strain using a stable GT. Importantly, identification of outbreaks should not rely on GTs alone but should also consider closely related clusters (e.g. ODCs) and epidemiological data.

Because the first seven MGT levels were mutually exclusive, hierarchical inconsistency can occur where mutations in low resolution levels lead to two STs that are assigned the same ST in a higher resolution level. This issue can be resolved in two ways. Firstly, overall relatedness of two isolates should be measured by the largest level at which they share an ST. Work is currently underway to develop methods to automatically correct hierarchical inconsistency on this basis. For example, if two isolates differ at MGT4 but have the same ST at MGT5 the MGT5 ST should be used to describe the relationship between the isolates. It should be noted that if the MGT levels were nested rather than mutually exclusive there would be no mechanism to correct or even recognise this hierarchical inconsistency. Secondly, CCs can be used to group isolates together that differ by only one allele in each level. In this way, the above scenario would be resolved as the two MGT4 STs that differ by a single allele would be clustered into the same CC. We have implemented this additional feature into our scheme for flexibility. However, CCs should be used alongside degenerate GT assignment due the instability of CCs.

MGT will be useful in tracking clones of varying levels of virulence and antibiotic resistance. Clones can be defined by the MGT level with optimal resolution for the application at hand. This flexibility will allow precise identification and tracking of strains displaying traits of particular public health significance such as multidrug-resistance and hypervirulence.

MGT has been tested on STM as an example and further studies will be required to determine the applicability of the MGT to a broad range of species with varying population structures and degrees of clonality. However, the principles used to design this scheme should be applicable to a large number of bacterial species and clones (especially those with mostly clonal populations). Our preliminary testing of MGT on another *Salmonella* serovar, *S*. Enteritidis and another species, *Vibrio* cholerae, suggest the MGT concept is applicable in these contexts; however, highly recombinogenic clones/species have yet to be examined. MGT empowers simple and concise communication of genetic relationships between isolates in a simple string of numbers and could provide a universal naming system for a given species or clone.

## Conclusion

MGT provides a promising solution for genomic nomenclature of STM strains and could be implemented as an internationally standardised strain identification system that is suitable for both long-term and short-term epidemiology. Further work in a wide range of species with different population structures is required to examine the general applicability of MGT. With further refinement, MGT has the potential to provide a widely applicable, stable nomenclature system for different bacterial species.

## Availability of data and materials

All raw sequencing data used in this study is available from the sequence read archive (SRA) or the European nucleotide archive (ENA) using the accessions listed in Supplementary Table 10. Custom scripts used in this study are available at https://github.com/LanLab/MGT.

## Supplementary Data

165000 Supplementary Methods

## Supplementary Data

245000 Supplementary Figures

## Supplementary Data

3191000 Supplementary Tables
