# Supplementary material for: Multilevel genome typing: genomics-guided scalable resolution typing of microbial pathogens
Source: Euro Surveill. 2020 May 21;25(20):1900519. doi: 10.2807/1560-7917.ES.2020.25.20.1900519 (PMC7262494; doi:10.2807/1560-7917.ES.2020.25.20.1900519)
Supplement: Supplementary Methods [file 1900519_LAN_Supplementary_Methods.pdf]

This supplementary material is hosted by Eurosurveillance as supporting information alongside the article "Multilevel genome typing: genomics guided scalable resolution typing of microbial pathogens" on behalf of the authors, who remain responsible for the accuracy and appropriateness of the content. The same standards for ethics, copyright, attributions and permissions as for the article apply. Supplements are not edited by Eurosurveillance and the journal is not responsible for the maintenance of any links or email addresses provided therein.

Supplementary methods

Scripts

All code used to run MGT analysis is available at https://github.com/LanLab/MGT. Additional information including data submission and QC is available at https://mgt-docs.readthedocs.io/en/latest/.

Data submission and QC

Data submission is available through the mgtdb.unsw.edu.au site once an account has been set up and the user has logged in. Both publicly visible and private isolate modes are available. QC is handled by an automated pipeline with genome assembly filters specific for each database. The details of the pipeline are available at https://mgt-docs.readthedocs.io/en/latest/analysis_pipeline.html#reads-to-alleles. Briefly, kraken is used to verify that the reads belong to the correct species and that no minor contamination exists [1]. The shovill genome assembly pipeline (http://github.com/tseemann/shovill) is then used with SKESA as the assembler [2]. Shovill includes several read quality and correction steps. The completed assembly is then compared to species specific genome quality filters mentioned above. Finally SISTR is used to verify the serovar of the *Salmonella* isolate [3]. Further details of the entire MGT calling pipeline are available at https://mgt-docs.readthedocs.io/en/latest/analysis_pipeline.html#. To facilitate rapid data upload part of this pipeline can be carried out locally and the resulting fasta file uploaded instead of the larger fastq raw read files. The partial pipeline and installation instructions can be found at https://github.com/LanLab/MGT_reads2alleles.

Scheme definition

Scheme size selection using mutation rates

In order to define sizes of schemes so that they will describe timeframes that are relevant to epidemiological studies the sizes of schemes 2 to 7 were determined using the average mutation rate for STM. The estimated STM mutation rate from multiple studies were averaged to produce the rate of 1.03E-06 mutations per site per year [4-9] which corresponds to approximately 4.62 mutations per genome per year. This rate was used to estimate what proportion of the genome would be required for a given scheme to gain one mutation every X years. For example for MGT7 this timeframe was 1 year. One mutation per year is equivalent to a rate 1/4.62 as fast as the genome as a whole and therefore the scheme was selected to be 1/4.62 the length of the genome. The LT2 STM genome is 4,857,450bp long therefore the MGT7 scheme size target was 1,051,647bp. This process was repeated for MGT6, 5, 4, 3 and 2 with age targets of 2, 5, 10, 20 and 100 years respectively.

Assignment of loci to MGT levels based on locus characteristics and relative genome position

Multiple characteristics of each locus were examined in order to identify which loci should go into which scheme. The overriding goal of this process was to ensure that the smaller schemes reflected the overall characteristics of the genome as closely as possible, which would ensure that the overall mutation rate of these schemes would be likely to maintain the mutation rate per position of the genome as a whole. In smaller schemes if one locus is evolving very quickly due to selective pressure this will distort the rate of ST formation for the whole level. However in larger schemes the volume of loci means that the impact of any one locus is reduced. The DnDs value for each locus was examined using data from a previous study [10] and loci between the first and third quartiles was initially used in MGT2 followed by loci from the 5th to 95th percentiles for MGT 3-7. The reliability of intact allele calling was also taken into account to ensure that smaller schemes were assigned the most reliable loci. 9799 genomes were processed, and a locus was included in MGT2,3 and 4 if it was never called as missing or partially missing, this was reduced to allow a maximum of 5 for each type for MGT5 and 6 and subsequently further reduced to 25 for MGT7. It should be noted that even at 25 genomes allowed missing or partially missing loci this amounts to 0.025% of genomes missing in the most unreliable loci. An Enterobacteriaceae core was defined using 20 species (supplementary table 6) using roary ([11], v3.12.0) with sequence identity of 70% and presence proportion of 100% and included 1540 loci. Only loci from this core were included in MGT2 and MGT3. TMHMM, signalP, biocyc and psort were used to classify loci into their subcellular locations and loci encoding transmembrane, cell surface, cell wall or secreted proteins were excluded from MGT2, 3 and 4. Loci were also excluded from MGT2-6 when they matched any of the following criteria: phage encoded genes (as predicted by PHASTER [12]); loci containing homopolymers longer than 8bp; loci containing tandem repeats (defined by tandem repeat finder [13]).

In addition to these filters a minimum distance between each locus included in a given scheme was defined to reduce any potential impact from recombination. These minimum distances are listed in supplementary table 8. Importantly these distances are also relative to loci included in previous levels. For example, if a locus is assigned in MGT4 it will be a minimum of 4Kb from any locus included in MGT2, 3 or 4.

Missing Data

Alleles

Each MGT level sequence type is defined by an allele profile which is in turn made up of allele calls for individual loci. If a locus matches an existing allele exactly or is new but has no missing data it can be assigned a positive allele (i.e. 6) indicating that it is intact and there is no uncertainty about its identity. If a locus is missing more than 20% of its sequence it is assigned a 0 allele (missing) and no inference about its relationships to other alleles is attempted. Alleles that have no genetic differences from an intact allele but are missing less than 20% of their sequence are termed ‘negative alleles’. For example, when a new allele is missing 5% of its length the remaining 95% is compared to other intact alleles of that locus. If there is another intact allele with no SNP differences from the new allele, the new allele is assigned as a negative version of the positive allele (i.e. intact allele 4 vs partially missing negative allele -4_1). In this way as much genetic information as possible is retained within the negative allele. One intact allele (e.g. 4) can have multiple negative alleles which differ from each other only in the location of the missing data they contain (e.g. -4_1 and -4_2). A negative allele can only be assigned to a locus with missing data if an intact, matching allele

exists. If no intact allele exists, then the negative allele is called as a 0. In cases where a new negative allele matches to more than one positive allele the frequency of the positive alleles in the database subset (see database subsetting section) are examined and the allele is assigned to the most frequently occurring positive allele in the subset. Importantly a negative allele will not cause a new ST to be assigned if it is the only difference between an existing allele profile (locusX allele of 4) and a new allele profile (locusX allele of -4_2).

Sequence types

For each MGT level the allele profile of the new isolate is compared to existing profiles. If all loci in an allele profile match then the new isolate is assigned the corresponding ST at that level. If there is a difference in one or more loci to all existing profiles that is not due to missing data then a new ST is assigned. If an allele profile is defined with missing data (either zero or negative allele calls) it is assigned a degenerate sequence type (dST) this dST is combined with the ST to produce an ST assignment that communicates both the known genetic information and also the fact that there is some uncertainty involved. A single ST (e.g. MGT8 ST234) can have multiple dSTs that differ by having missing data in different loci. For example MGT8 ST234.1 and MGT8 ST234.2 have the same allele assignments but differ by having negative alleles at different loci. Because these dSTs can have missing loci there is a small chance that two intact STs will match to the same dST. In this situation the dST is assigned to the ST that is most prevalent in the database subset (see database subsetting section). If more than a given threshold of loci have zero alleles (for *Salmonella* Typhimurium this was 2%) the ST will not be called and a blank will be entered into the database.

Database subsetting

During processing STs and CCs are identified for one scheme before moving on to the next largest scheme (i.e. MGT2 ST and CC are assigned before allele calling for MGT3 starts). This allows results from smaller schemes to restrict the search space for larger schemes. This is done by only searching allele profiles found in isolates that share a CC with the strain being analysed. For example a new strain has been assigned MGT2 CC3 and MGT3 CC23. When the analysis gets to MGT6, alleles found in other isolates that are in those same MGT2 and 3 CCs will be compared to the new strain. This subsetting of the possible MGT6 alleles and STs significantly reduces running time for larger schemes. Additionally, because this method groups related isolates together we can use the occurrence of alleles and STs within the subset as a tiebreak for uncertain allele and ST calls.

1. Wood DE, Salzberg SL. Kraken: ultrafast metagenomic sequence classification using exact alignments. Genome Biol. 2014;15(3):R46.
2. Souvorov A, Agarwala R, Lipman DJ. SKESA: strategic k-mer extension for scrupulous assemblies. Genome Biol. 2018;19(1):153.
3. Yoshida CE, Kruczkiewicz P, Laing CR, Lingohr EJ, Gannon VP, Nash JH, et al. The Salmonella In Silico Typing Resource (SISTR): An Open Web-Accessible Tool for Rapidly Typing and Subtyping Draft Salmonella Genome Assemblies. PLoS One. 2016;11(1):e0147101.
4. Octavia S, Wang Q, Tanaka MM, Sintchenko V, Lan R. Genomic heterogeneity of Salmonella enterica serovar Typhimurium bacteriuria from chronic infection. Infection,

genetics and evolution : journal of molecular epidemiology and evolutionary genetics in infectious diseases. 2017;51:17-20.
5. Octavia S, Wang Q, Tanaka MM, Sintchenko V, Lan R. Genomic Variability of Serial Human Isolates of Salmonella enterica Serovar Typhimurium Associated with Prolonged Carriage. Journal of clinical microbiology. 2015;53(11):3507-14.
6. Okoro CK, Kingsley RA, Connor TR, Harris SR, Parry CM, Al-Mashhadani MN, et al. Intracontinental spread of human invasive Salmonella Typhimurium pathovariants in sub-Saharan Africa. Nat Genet. 2012;44(11):1215-21.
7. Okoro CK, Barquist L, Connor TR, Harris SR, Clare S, Stevens MP, et al. Signatures of adaptation in human invasive Salmonella Typhimurium ST313 populations from sub-Saharan Africa. PLoS Negl Trop Dis. 2015;9(3):e0003611.
8. Mather AE, Reid SW, Maskell DJ, Parkhill J, Fookes MC, Harris SR, et al. Distinguishable epidemics of multidrug-resistant Salmonella Typhimurium DT104 in different hosts. Science. 2013;341(6153):1514-7.
9. Hawkey J, Edwards DJ, Dimovski K, Hiley L, Billman-Jacobe H, Hogg G, et al. Evidence of microevolution of Salmonella Typhimurium during a series of egg-associated outbreaks linked to a single chicken farm. BMC Genomics. 2013;14:800.
10. Desai PT, Porwollik S, Long F, Cheng P, Wollam A, Bhonagiri-Palsikar V, et al. Evolutionary Genomics of Salmonella enterica Subspecies. MBio. 2013;4(2).
11. Page AJ, Cummins CA, Hunt M, Wong VK, Reuter S, Holden MT, et al. Roary: rapid large-scale prokaryote pan genome analysis. Bioinformatics. 2015;31(22):3691-3.
12. Arndt D, Grant JR, Marcu A, Sajed T, Pon A, Liang Y, et al. PHASTER: a better, faster version of the PHAST phage search tool. Nucleic Acids Res. 2016;44(W1):W16-21.
13. Benson G. Tandem repeats finder: a program to analyze DNA sequences. Nucleic Acids Res. 1999;27(2):573-80.
