# Supplementary material for: Multilevel genome typing: genomics-guided scalable resolution typing of microbial pathogens
Source: Euro Surveill. 2020 May 21;25(20):1900519. doi: 10.2807/1560-7917.ES.2020.25.20.1900519 (PMC7262494; doi:10.2807/1560-7917.ES.2020.25.20.1900519)
Supplement: Supplementary Figures [file 1900519_LAN_Supplementary_Figures.pdf]

This supplementary material is hosted by Eurosurveillance as supporting information alongside the article “Multilevel genome typing: genomics guided scalable resolution typing of microbial pathogens” on behalf of the authors, who remain responsible for the accuracy and appropriateness of the content. The same standards for ethics, copyright, attributions and permissions as for the article apply. Supplements are not edited by Eurosurveillance and the journal is not responsible for the maintenance of any links or email addresses provided therein.

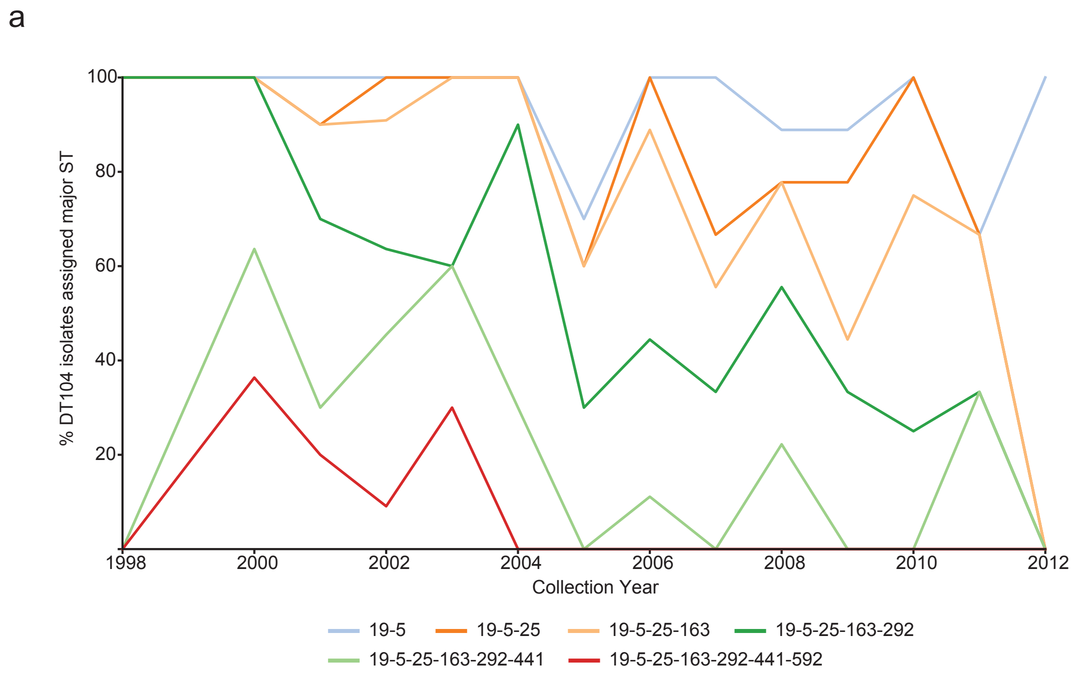

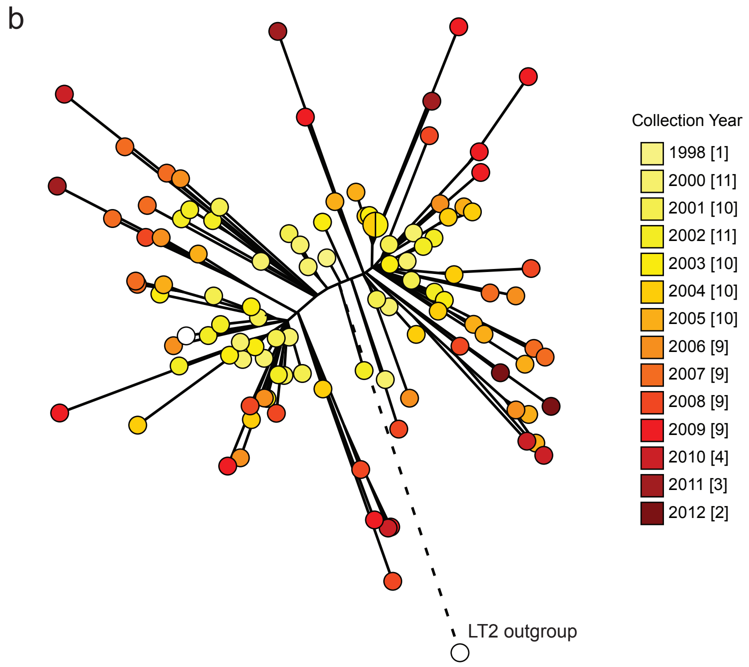

Supplementary Figure 1. Temporal increase in diversity of DT160 isolates from New Zealand point source epidemic. a. The proportion of 109 isolates sequenced in Bloomfield et al assigned to the most common MGT2-7 STs over time. The major ST for each level was determined and the percentage of isolates in a given year classified into that ST is shown. With the exception of MGT2 and 3 all other MGT levels show a reduction on the major type over time. **b.** Relatedness tree generated from MGT9 allele profiles of 109 DT160 isolates with LT2 as an outgroup using Grapetree. Nodes are coloured by year with a clear temporal signal present as the initial source diversified over time.

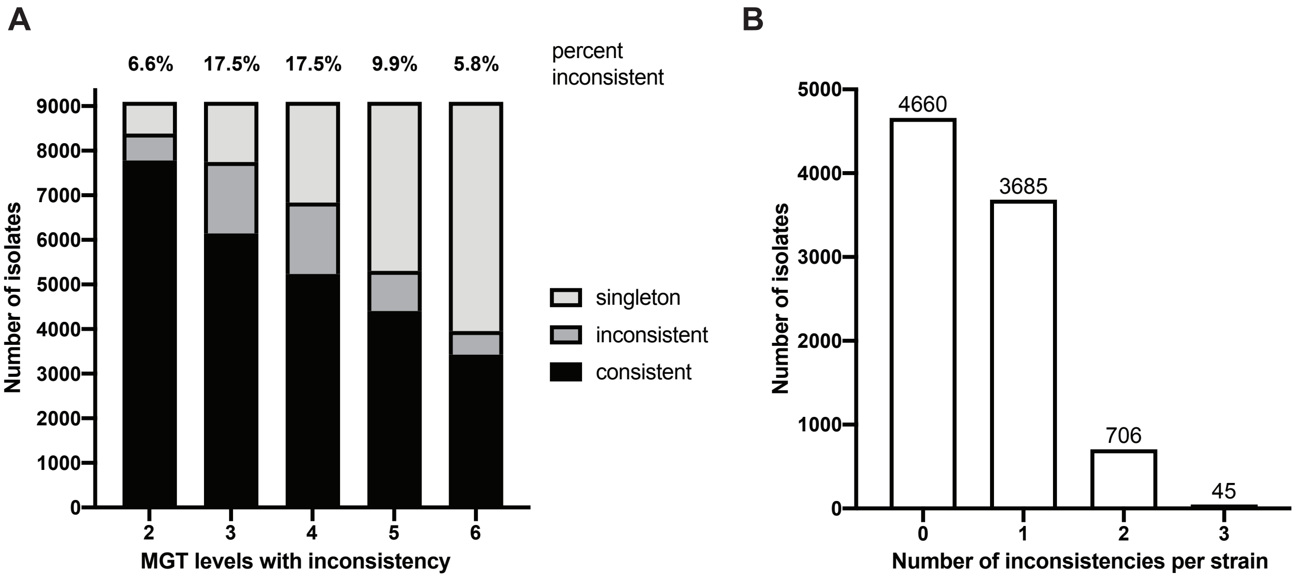

Supplementary figure 2. Hierarchical inconsistencies in STM isolates assigned MGT types. A. The number of isolates with with a GT that contains STs that are in the same at a larger level (e.g. MGT5) but different at a smaller level (e.g.MGT4). The smaller scheme is shown in the X axis. The percentage of isolates with an inconsistent GT at each level is shown above each column. Singletons have only one isolate in the larger level sequence type and so inconsistency cannot be evaluated. B. A count of the number of levels that are inconsistent in each isolate. The majority of isolates 4660 (51.2%) had no inconsistencies, 3685 (40.5%) had one, 706 (7.8%) had two and 45 (0.5%) had three.

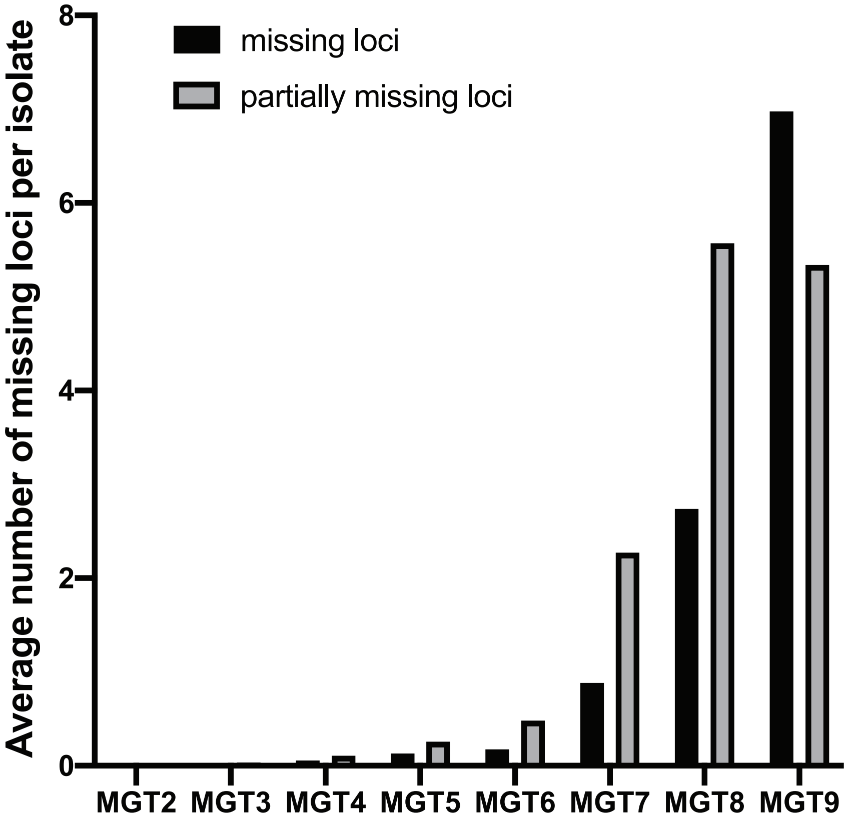

Supplementary Figure 3. Average number of missing and partially missing loci per isolate per MGT level. Partially missing is defined as missing between greater than 0 and 20% of a given locus. Missing is defined as missing greater than 20% of a given locus. Lower levels (MGT2-6) have very few partially or completely missing loci due to the selection of loci for these schemes that are most reliably called by the MGT typing scripts. Less reliable loci were placed in the largest schemes.
